# Enhancing Circular Polarization Performance of Low-Profile Patch Antennas for Wearables Using Characteristic Mode Analysis

**DOI:** 10.3390/s23052474

**Published:** 2023-02-23

**Authors:** Zhensheng Chen, Xuezhi Zheng, Chaoyun Song, Jiahao Zhang, Vladimir Volskiy, Yifan Li, Guy A. E. Vandenbosch

**Affiliations:** 1 Division ESAT-WaveCoRE, KU Leuven, 3001 Leuven, Belgium; 2 Department of Engineering, Strand Campus, King’s College London, London WC2R 2LS, UK; 3 National Key Laboratory of Science and Technology on Vessel Integrated Power System, Naval University of Engineering, Wuhan 430000, China

**Keywords:** characteristic mode, parasitic loading, slit loading, low-profile, wearable antenna

## Abstract

A wearable antenna functioning in the 2.4 GHz band for health monitoring and sensing is proposed. It is a circularly polarized (CP) patch antenna made from textiles. Despite its low profile (3.34 mm thickness, 0.027 *λ*_0_), an enhanced 3-dB axial ratio (AR) bandwidth is achieved by introducing slit-loaded parasitic elements on top of analysis and observations within the framework of Characteristic Mode Analysis (CMA). In detail, the parasitic elements introduce higher-order modes at high frequencies that may contribute to the 3-dB AR bandwidth enhancement. More importantly, additional slit loading is investigated to preserve the higher-order modes while relaxing strong capacitive coupling invoked by the low-profile structure and the parasitic elements. As a result, unlike conventional multilayer designs, a simple single-substrate, low-profile, and low-cost structure is achieved. While compared to traditional low-profile antennas, a significantly widened CP bandwidth is realized. These merits are important for the future massive application. The realized CP bandwidth is 2.2–2.54 GHz (14.3%), which is 3–5 times that of traditional low-profile designs (thickness < 4 mm, 0.04 *λ*_0_). A prototype was fabricated and measured with good results.

## 1. Introduction

Due to the restrictions on cost, reliability, productivity, safety, and robustness, as well as the requirement for a good user experience in advanced communications and wireless body area networks (WBAN) for health care, health monitoring, sensing, and athlete training, etc., a tremendous effort has been devoted to minimizing traditional rigid antennas [1,2]. However, as a compromise, the miniaturization sacrifices the antenna performance since the antenna gain is essentially related to its dimensions [3]. Alternatively, flexible wearable antennas based on textiles [4,5,6], polyimide [7], PDMS [8,9,10], etc., are supposed to be good candidates for maintaining good performance and user experience while relieving the dimension restriction. For instance, [6] reports a wearable antenna for energy harvesting applications; however, the reported antenna has a low front-to-back ratio (FBR) without ground shielding. It is well known that the unshielded design results in a significant gain decrease (e.g., it decreases by a factor of 2 or more in [6]) due to the coupling and power absorption caused by the vicinity loading of the lossy human body. In contrast, patch antennas are reported to be insensitive to the human body and can obtain high FBR due to their shielding ground, which can benefit wearable applications. Their merits of low profile and low cost are also favorable for massive usage. Additionally, one needs to address the issue of polarization mismatch, which inevitably happens due to unpredictable movements in realistic circumstances when linearly polarized (LP) antennas are used. Hence, CP antennas are a more appropriate choice [11,12,13,14,15,16]. Ref. [11] reports an S-shape slot CP antenna with a narrow 3-dB AR bandwidth (1.23%) for Iridium and GPS communications. Ref. [12] presents an omnidirectional button antenna using conceptual magnetic dipoles for 5 GHz applications. There are some other wearable CP antennas designed using a low-profile approach since clothes mostly have a thin thickness [17,18,19,20,21,22]. However, according to the above-mentioned literature, most of these wearable antennas have a limited CP bandwidth and a degraded performance in harsh environments. There are some antennas with bandwidth-enhancing techniques reported for traditional rigid antenna designs, for example, using aperture coupling [23], metamaterials/metasurfaces [24,25,26,27,28], parasitic elements [14,23], thick-air substrates [14,24], multilayer stacking [13], pin-loading [29], reflectors (e.g., AMC, PRS) [30,31], AI-based approaches [25,32], etc. However, most of these popular techniques are not suitable for wearable applications because of the thickness restrictions.

In this paper, we present a low-profile slit-loaded CP antenna with enhanced 3-dB AR bandwidth using Characteristic Mode Analysis (CMA). Its CP bandwidth is 2.2–2.54 GHz (14.3%). This design features two essential merits over the literature: (1) a superior CP bandwidth and high gain are achieved for low-profile wearable CP antennas in the 2.4 GHz band; (2) increased bandwidth with only single layer substrate, which is easy to fabricate and low-cost. It is worth mentioning that not many designs are capable of obtaining a decent 3-dB AR bandwidth for low-profile CP antennas (LPCPA), as depicted in Table 1, whereas our work draws a clear distinction. In detail, a multilayer structure with stacked patches and parasitic elements is reported in [13], which leads to 20.7% CP bandwidth at 6 GHz. Ref. [14] reports a strip-loaded antenna with 24% CP bandwidth. However, they are only suitable for traditional rigid substrates. Ref. [17] presents a wearable antenna array fed by a sequential feeding network. The feeding is embedded in the inner layer of the antenna, which makes the fabrication complicated. A modified metasurface is also applied to the antenna design in [18], which results in a wideband property with a peak gain of 8.5 dBic. In [12,19], shorting pins are used to design flexible antennas for off-body or on-body communication. However, these antennas work in the 5 GHz band [12,13,17,18,19]. If such antennas are scaled to the 2.4 GHz band, the physical thickness of the antennas increases. The scaled designs may be too bulky for wearable applications. As for the 2.4 GHz band low-profile wearable designs [16,20,21,22], they all have a limited CP bandwidth. This is due to the fact that with a physically similar low-profile thickness, their electrical thickness in the 2.4 GHz band is smaller than that one in the 5 GHz band, and thus, leads to a high Q, in other words, a narrow bandwidth in the 2.4 GHz band. Comparing to these reported wearable designs with a low profile, the proposed antenna achieves the highest CP bandwidth of 14.3%, around 3–5 times that of the counterparts. This is accomplished by adopting parasitic elements, as in [14], and a novel slit-loading technique. Notably, except for the parasitic loadings, Ref. [14] also utilizes a widely used bandwidth boosting method, i.e., air gap loading [14,23], and two blind disk-loaded pins to realize a single-feed design. However, the approach in [14] is unfavorable for wearables because of the thick air gap, and the blind disk and pins make the antenna fabrication complicated and costly (which are not easy to be addressed for wearables). Moreover, it is worth emphasizing that unlike traditional parasitic loadings [14,29], the bandwidth enhancement in this work is also attributed to the slit-loading approach, which introduces higher-order modes and also unblocks the requirement of inductive matching compensation in traditional designs. Overall, both the parasitic elements and the slit-loading lead to a decent CP bandwidth with a low-profile single-layer substrate.

This work is organized as follows. Section 2 illustrates the antenna configuration. Section 3 introduces the CP bandwidth-enhancing procedure from the perspective of CMA. Further, under the guidance of the obtained CMA results, CST microwave studio 2020 is employed to validate the antenna design with full-wave simulations. Then the antenna is fabricated, and the measurement results are discussed in Section 4. Finally, conclusions are given in Section 5.

## 2. Antenna Configuration

**Figure 1:**
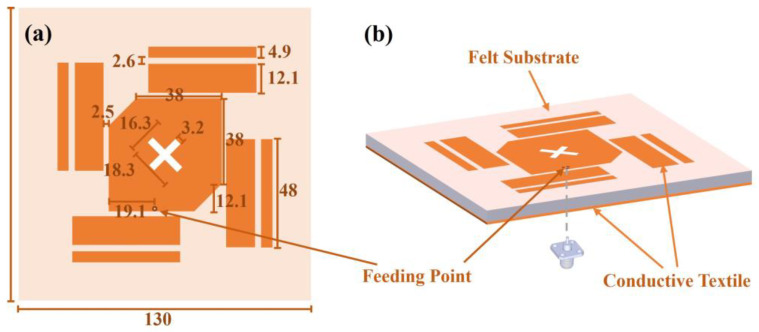
Structure of the antenna. (**a**) Front view. (**b**) 3D view.

The geometry of the antenna is depicted in Figure 1. The antenna is composed of a corner-truncated patch surrounded by parasitic elements. The patch is with a cross-slot at the center. The antenna is deposited on top of a grounded single substrate layer made of felt ($\varepsilon_{r}$ = 1.35, tanδ = 0.03). Both the patch and the ground are made of conductive textiles with a conductivity of *σ* = 1.18 × 10^5^ S/m (Shieldit Super from LessEMF Inc, Latham NY, USA.). The materials are flexible so that the antenna is friendly to be worn on the body. A ground shielding topology rather than unshielded ones [6,33] is adopted to reduce the human body loading effect on the antenna performance. Since a large area is available on the body, the antenna ground can be kept large to have higher gain and less radiation exposure issues. The thickness of the felt substrate and the conductive textiles are 3 mm and 0.17 mm, respectively. This results in a low-profile structure whose thickness is only 0.027 $\lambda_{0}$. An antenna prototype is fabricated, as shown in Figure 9a. All the materials were cut manually. The textile radiator, the ground layer, and the felt substrate were ironed together with thermal melting glue. An SMA connector was soldered to the ground and to the patch to feed the antenna for measurements using a temperature-tunable soldering iron to avoid burning the textile.

## 3. Working Mechanism

### 3.1. Characteristic Mode (CM) Theory

To ease the illustration of the working mechanism of the CP bandwidth enhancement, here we review the main theoretical elements of CMA first.

According to the CM theory [34], an impedance matrix Z can be written as

Z=R+jX,

where R and X are the real and imaginary parts of the impedance matrix, respectively. Accordingly, a generalized eigenvalue problem is defined as:

$$XJ_{n}=\lambda_{n}RJ_{n},$$

where $J_{n}$ is the *n*th characteristic current mode of a conductive structure and $\lambda_{n}$ is the corresponding eigenvalue of the *n*th mode. For each mode, modal significance MS is defined as [35]:

$$\mathrm{MS}=\left|1/\left(1+j\lambda_{n}\right)\right|.$$

It is an intrinsic property of the structure and is independent of external excitations. Thus, by investigating the MSs of a certain structure, the properties of the structure can be envisioned and modified. Especially for $\lambda_{n}\;$= 0, i.e., MS = 1, the *n*th mode is in resonance. If we take an antenna as an example, with proper feeding excitation, the antenna may effectively radiate.

Another important factor for antennas design in CM theory is the characteristic angle (CA), which is defined as [35]:

$$\alpha_{n}=180^{o}-\tan^{-1}\lambda_{n}.$$

It presents the constant phase lag between the current $J_{n}$ and the tangential component of characteristic fields $E_{n}$, where $E_{n}$ is the field produced by current $J_{n}$.

To design a CP antenna, in practice, it is required that two orthogonal modes be effectively excited, and their MSs be close. Meanwhile, the characteristic angles should have a phase difference of nearly 90° (70°–110°).

### 3.2. Characteristic Mode Analysis (CMA) of the Low-Profile CP Antenna

**Figure 2:**
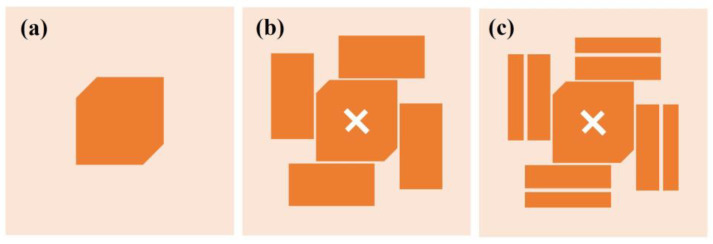
Evolution of the LPCPA design. (**a**) Step 1, a traditional corner truncated patch antenna. (**b**) Step 2, a corner truncated patch with cross-slot and parasitic elements. (**c**) Step 3, the proposed LPCPA with slit-loading.

**Figure 3:**
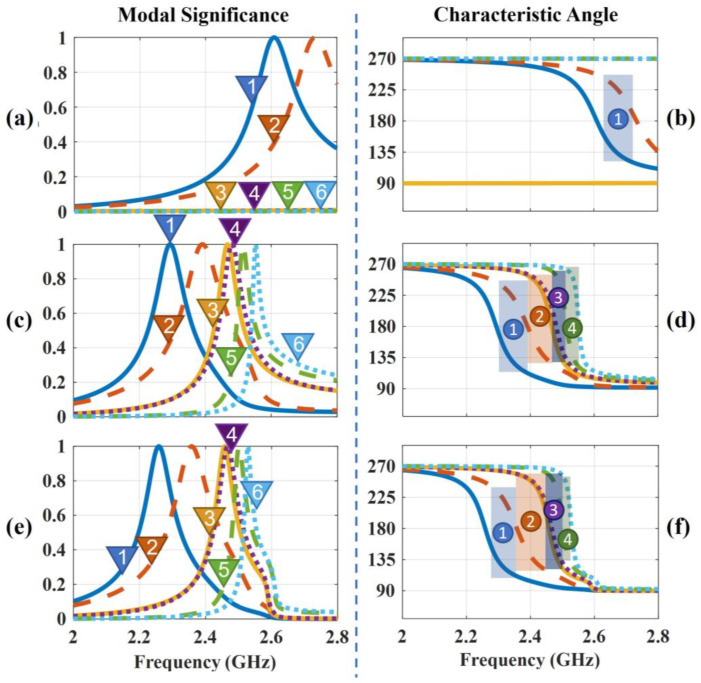
MSs and CAs evolution of the LPCPA. (**a**,**b**) MSs and CAs of Step 1. (**c**,**d**) MSs and CAs of Step 2. (**e**,**f**) MSs and CAs of Step 3. Colored zones present where the CA phase differences are in the range of 70°–110°.

**Figure 4:**
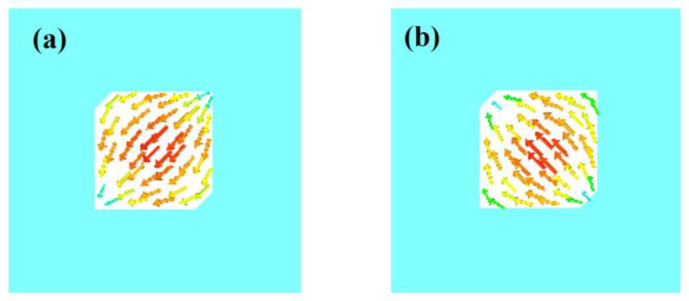
Modal current distributions of basic corner-truncated structure. (**a**) Mode 1. (**b**) Mode 2.

**Figure 5:**
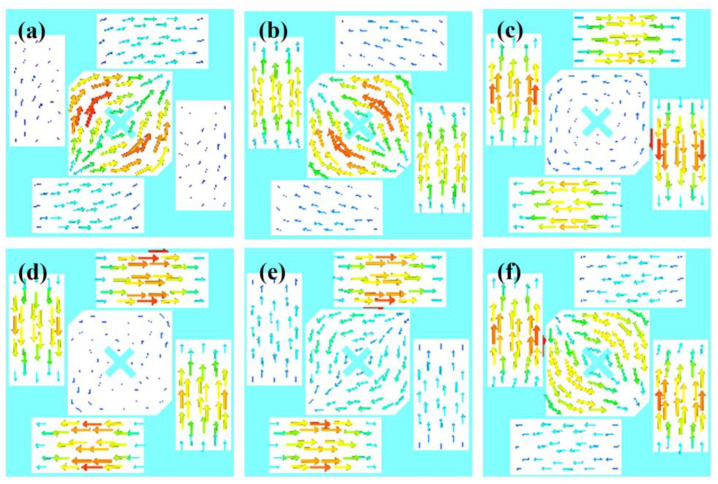
Modal current distributions of the structure without slit-loading. (**a**–**f**) are Mode 1–6.

**Figure 6:**
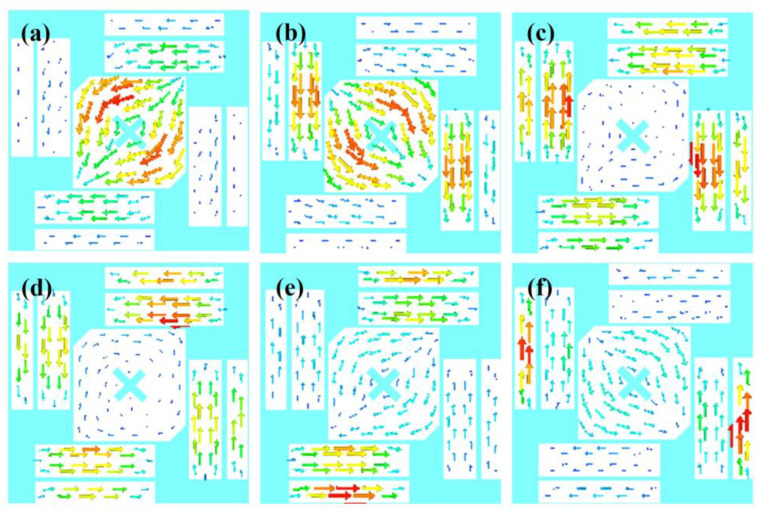
Modal current distributions of the structure with slit-loading. (**a**)–(**f**) are Mode 1–6.

CMA Multilayer Solver in CST studio 2020 is applied to carry out the predesign and analysis of the proposed antenna. Figure 2 shows the design in 3 steps. The design starts from a traditional corner-truncated patch, and then a cross-slot and four parasitic elements are adopted to introduce extra resonant modes in the frequency band of interest. Afterward, slits are cut on the parasitic elements to further broaden the bandwidth. Figure 3 illustrates the MSs and CAs of the first six modes of the three-step design, and Figure 4, Figure 5 and Figure 6 illustrate the corresponding modal current distributions. As we can see in Figure 3a, mode 1 and mode 2 have large MSs in the 2.5–2.8 GHz band, while the MSs of mode 3–6 are almost 0, which means mode 1 and mode 2 could be excited. However, the MS and CA conditions to generate CP waves are effective in a limited bandwidth, i.e., 2.62–2.72 GHz, indicated in the colored zone 1 in Figure 3b. This also explains the natural narrow CP bandwidth of traditional corner-truncated patch antennas [21,22,36]. 

To increase the bandwidth, four parasitic elements are utilized to introduce extra modes in the interested band in the second step. The MSs and CAs for the interested mode 1–6 are shown in Figure 3c,d. It can be seen that mode 1 and mode 2 are the dominant ones and satisfy the CA requirements for generating CP waves around 2.34 GHz (colored zone 1, 2.3–2.39 GHz), while other modes around 2.34 GHZ have low MSs so that they can be ignored. Similarly, mode 2 and mode 3/4 contribute to a potential CP around 2.45 GHz (colored zone 2), mode 3/4 and mode 5 contribute to the frequency band of 2.47–2.51 GHz (colored zone 3), and mode 5 and mode 6 contribute to 2.51–2.55 GHz (colored zone 4). To sum up, the antenna may theoretically have a CP bandwidth in the frequency band of 2.3–2.55 GHz as long as all the modes can be effectively excited. Notably, the resonant frequencies of mode 1 and mode 2 are lower than the ones in step 1. This is due to the introduction of the cross-slot at the patch center and due to the coupling between the parasitic elements and the patch. This can be seen from the modal current distributions of mode 1 and mode 2, as shown in Figure 4a,b and Figure 5a,b. Such coupling can be modeled using LC model methods [5,28]. Moreover, it is worth mentioning that the parasitic patches and the traditional center patch without slot-loading in step 1 induce a strong capacitive effect with the antenna ground. This makes a traditional high-profile design preferable since a long feed probe can be used to compensate for the capacitive effect by its intrinsic inductance. However, for a wearable design with low-profile restrictions, the feed probe is too short to fulfill this requirement. Hence, a cross-slot is cut at the center patch to reduce the capacitive effect and to release the need for a long probe. 

Last, we find that the topology with slit-loadings on the parasitic elements proposed in step 3 has similar MSs, CAs, and modal currents as in step 2 (see Figure 3e,f and Figure 6). The potential CP bandwidth is 2.26–2.52 GHz. This is due to the fact that all the modal currents of modes 1–6 on the parasitic elements flow along the *x* or *y* direction so that slits along *x* or *y* directions have limited impact on the modal currents and the characteristic properties. Thanks to this, narrowing down the size of parasitic patches in step 2 by means of additional slits increases the parasitic inductance and decreases the capacitance while keeping the characteristic properties, meaning that a short, less inductive feeding structure may be sufficient to feed and excite the antenna. Overall, this approach with slits and the cross-slot is significantly different from the thick multilayer designs in [14,29], which need extra disks and pins for impedance matching.

### 3.3. Full-Wave Simulation

**Figure 7:**
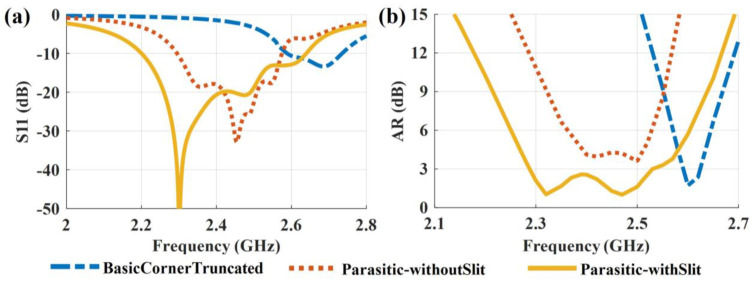
Simulated (**a**) S11 and (**b**) AR evaluation of the antennas.

Summarized, topology 3 has the potential to obtain a wide CP bandwidth if the desired modes 1–6 are effectively excited with proper feeding. In the following, a coaxial probe (inner-pin diameter is 1 mm, outer-shell diameter is 4.1 mm) is used as feeding, and full-wave simulations are carried out to validate the proposed design. The final optimized parameters are given in Figure 1. The simulated reflection coefficient and AR results of the three topologies are shown in Figure 7. The full-wave simulation results agree with the above-analyzed results very well. It is clear that the proposed LPCPA with slit-loadings has significantly improved the performance of the low-profile antennas of topology 1 and topology 2. Its impedance bandwidth (S_11_ < −10 dB) and CP bandwidth (AR < 3 dB) are 2.2–2.64 GHz and 2.29–2.53 GHz, respectively.

**Figure 8:**
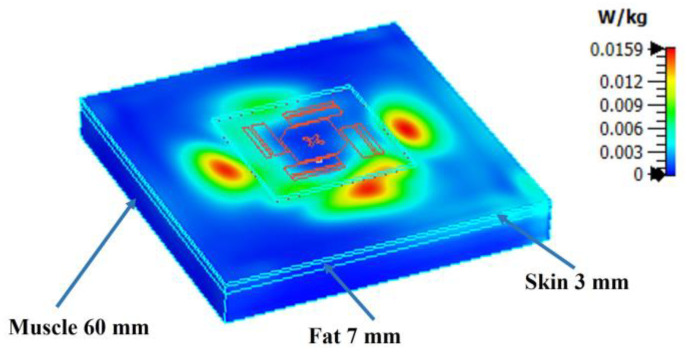
SAR simulation result at 2.4 GHz.

The Specific Absorption Rate (SAR) over 10 g of tissue is simulated by CST Studio 2020 according to the IEEE C95.3 standard. For realistic wearing applications, the antenna is placed about 5 mm above the body most of the time due to the insertion of air gaps and mid-layers to prevent scratching, potential allergic reactions, etc. Figure 8 shows the simulation model, which has been validated in [37,38]. The permittivity of the fat, skin, and muscle is 5.28, 38.01, and 52.73, respectively [39]. The simulated SAR result is 0.0159 W/kg at 2.4 GHz. The simulated SAR value is significantly lower than the European standard threshold of 2 W/kg. This is a benefit of the ground shielding of the proposed design.

## 4. Measurement and Discussion

**Figure 9:**
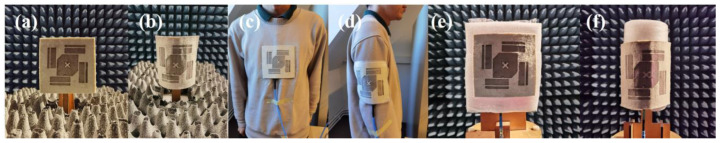
Measurement setups. (**a**,**b**) Free space. (**c**,**d**) On-body. (**e**,**f**) On-phantom.

The fabricated antenna was measured in an anechoic chamber with a Vector Network Analyzer 8510 system. Three scenarios were considered: free-space, on-body, and on-phantom. The measurement setups are shown in Figure 9. Since a wearable antenna is inevitably bent when being worn on the body in practice, bent antennas with curvature radii of 5 cm and 8.5 cm (i.e., conformal situations) were measured for the free space scenario. Further, the reflection coefficient of on-body scenarios, for example, on-arm and on-chest, was measured. As for the AR measurements, body tissue simulating liquid MSL2450v2 [40] was used to mimic the body loading due to the fact that a human may move during the measurement and cause unstable transmissions and reflections. A cylinder with a 10 cm diameter and a liquid bag packed with a 10-mm-thick foam were used as phantoms to mimic the on-arm and on-chest situations, respectively.

### 4.1. Reflection Coefficient

**Figure 10:**
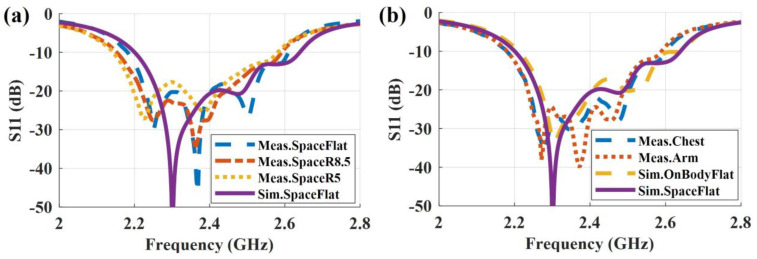
Simulated and measured reflection coefficient in (**a**) free-space and (**b**) on-body scenarios.

As shown in Figure 10a, in free space, the impedance bandwidth (S_11_ < −10 dB) is 2.2–2.64 GHz in simulation and 2.18–2.61 GHz in measurement when the antenna is flat. The slight discrepancy between simulation and measurement may be caused by manual fabrication errors. For the conformal cases, a slight frequency shift is observed. Nevertheless, the antenna still shows a good performance. Further, the on-body results are shown in Figure 10b. The simulated impedance bandwidth is 2.21–2.64 GHz, and the measured bandwidth of on-arm and on-chest is 2.18–2.59 GHz. It is evident that, due to the benefit of ground shielding, the proximity of the body results in a limited influence when the antenna is worn on the arm and on the chest. Overall, we can conclude that the simulation and the measurement results match well. The antenna shows a robust impedance performance in free space and on-body scenarios.

### 4.2. Axial Ratio

**Figure 11:**
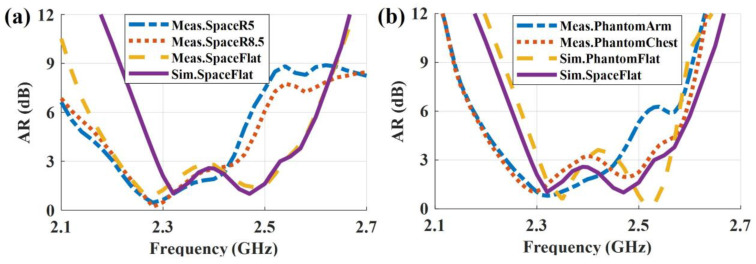
Simulated and measured AR in (**a**) free-space and (**b**) on-phantom scenarios.

The simulated and measured AR results of the antenna in free-space and on-phantom scenarios are shown in Figure 11. They are evaluated on the normal axis (*θ* = 0°) of the antenna. For the free-space scenario (see Figure 11a), the simulated flat antenna has a CP bandwidth (AR < 3 dB) of 2.29–2.53 GHz, and, accordingly, the measurement result is 2.2–2.54 GHz. The measurement bandwidth is wider than that of the simulation. This may be caused by fabrication errors during the manual fabrication process. The measured results when the antenna is bent show a frequency shift (depression) compared to the flat scenario. This is due to the symmetry of the structure that is broken. In detail, the modal currents of the dominant modes at high frequencies, i.e., modes 3–6 in Figure 6c–f, mainly flow on the rotationally symmetric parasitic elements. So, when the symmetric is broken, the CP waves cannot be effectively generated at high frequencies, and a frequency shift (depression) is observed. As for the on-phantom scenarios (see Figure 11b), a similar phenomenon can be observed.

### 4.3. Radiation Pattern and FBR

**Figure 12:**
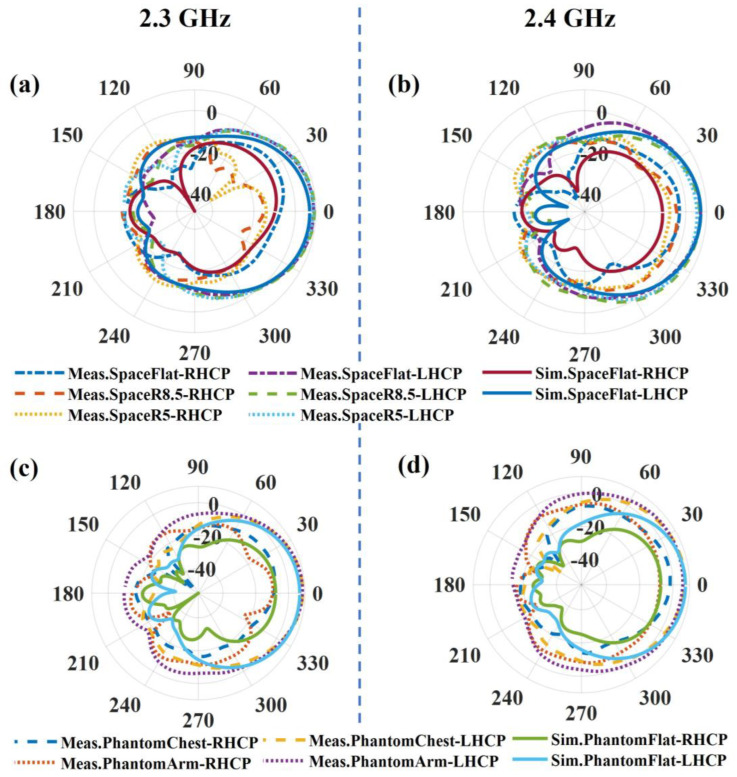
Simulated and measured realized gain pattern in (**a**,**b**) free-space and (**c**,**d**) on-phantom scenarios at 2.3 GHz and 2.4 GHz.

Figure 12 shows the LHCP and RHCP realized the gain pattern of the LPCPA at 2.3 GHz and 2.4 GHz for both the free space and the on-phantom scenarios. The LHCPs are 10 dB larger than the RHCPs, which means the main polarization of the antenna is left-handed circular polarization. In free space (Figure 12a,b), the flat antenna has a measured realized gain of 8.9 dBic and 7.1 dBic at 2.3 GHz and 2.4 GHz, respectively. Correspondingly, the estimated proximate efficiencies are 52.96% and 44.12% based on the half-power beamwidth [41]. Compared to the literature in Table 1, the designed low-profile antenna has achieved the highest peak gain even with lossy textile materials rather than the less-lossy PCB substrates that are used in [13,14]. Due to the bending, a decrease in the LHCP gain is observed. The measured realized gain is 8.7 dBic (2.3 GHz) and 5.4 dBic (2.4 GHz) when the antenna is bent with a curvature radius of 5 cm. As for the on-phantom scenario, see Figure 12c,d, the measured realized gains at 2.3 GHz are 8.6 dBic for on-chest and 8.4 dBic for on-arm, respectively, and at 2.4 GHz the corresponding ones are 7.1 dBic and 5.9 dBic. According to the patterns, we can also obtain the FBR values. The evaluated FBR values are all larger than 18 dB, and the peak one is 35 dB for the flat antenna in free space, which shows good radiation shielding due to the presence of the ground. As for positions having limited space or with drastic bending, for example, the wrist, the ground should be decreased accordingly for good wearing comfortability. Alternatively, the materials could be changed to more flexible and stretchable ones.

## 5. Conclusions

A wearable low-profile antenna with circular polarization is designed. Slit-loaded parasitic elements are used to improve the CP bandwidth in the 2.4 GHz band. CM theory is used to reveal the modal current distributions of the antenna. According to these current distributions, we find that the antenna can achieve a wide CP bandwidth by loading slits on the parasitic elements whilst the antenna profile remains low. Full wave simulations and measurements validate our design. The size of the prototype antenna is 1.04 × 1.04 λ_0_^2^ while the profile is only 0.027 λ_0_. The antenna has an impedance bandwidth of 2.18–2.61 GHz (18%) and a CP bandwidth of 2.2–2.54 GHz (14.3%) and realized a peak gain of 8.9 dBic. Compared to other low-profile wearable designs in the 2.4 GHz band, this design achieves a bandwidth that is 3–5 times larger.

## Figures and Tables

**Table 1:** Comparison with counterpart CP antennas in the literature.

Ref.	*f*_0_ (GHz)	Size (mm^3^, $\lambda_{0}\times \lambda_{0}\times \lambda_{0}$	Impedance BW (GHz)	3-dB AR BW(GHz)	Peak Gain	Efficiency	FBR (dB)	Wearable Material
[12]	5.8	14.1 × 14.1 × 10.29, 0.273 × 0.273 × **0.199**	5.68–5.91 3.97%	5.72–5.89 2.93%	2.1 dBic	72.6%	NA	Yes
[13]	**6**	40 × 40 × 4.5, 0.8 × 0.8 × **0.09**	5.0–7.0 33%	5.38–6.62 20.7%	8.6 dBic	NA	20 *	No, rigid
[14]	2.49	130 × 130 × 15, 1.079 × 1.079 × **0.125**	1.87–3.11 49.8%	2.32–2.95 **24%**	8.7 dBic	NA	25 *	No, rigid
[17]	5.44	40 × 40 × 4, 0.725 × 0.725 × **0.072**	4.25–6.63 43%	4.71–6.67 34%	7.5 dBic	NA	30 *	Yes
[18]	5.47	54 × 54 × 4.4, 0.985 × 0.985 × **0.080**	4.51–6.43 35.1%	5.02–5.98 17.5%	8.5 dBic	77%	35 *	Yes
[19]	5.86	35 × 35 × 2.24, 0.684 × 0.684 × **0.044**	5.67–6.05 6.6%	5.73–5.955 3.85%	7.2 dBic	NA	28 *	Yes
[20]	2.43	65.6 × 58.9 × 3.94, 0.531 × 0.477 × **0.032**	2.26–2.6 * 14.0%	2.365–2.499 **5.5%**	6.5 dBic	73%	25 *	Yes
[21]	2.45	100 × 100 × 3.94, 0.818 × 0.818 × **0.032**	2.34–2.57 9.4%	2.4–2.45 * **2.1%**	6 dBic	62%	30 *	Yes
[22]	2.5	60 × 60 × 3, 0.5 × 0.5 × **0.025**	2.36–2.64 11%	2.42–2.49 **2.86%**	1.8 dBic	30.7%	18 *	Yes
[16]	2.45	60 × 60 × 3.4, 0.48 × 0.48 × **0.027**	2.26–2.64 * 15.5%	2.3–2.4 **4.08%**	2.5 dBic	42.26%	25 *	Yes
**This work**	**2.4**	**130 × 130 × 3.34,** **1.04 × 1.04 × 0.027**	**2.18–2.61** **18%**	**2.2–2.54** **14.3%**	**8.9 dBic**	**52.96%**	**35**	**Yes**

NA: not available. *: evaluated from figures.

## Data Availability

Not applicable.
